# Development and characterization of sorafenib-loaded lipid nanocapsules for the treatment of glioblastoma

**DOI:** 10.1080/10717544.2018.1507061

**Published:** 2018-10-19

**Authors:** Anne Clavreul, Emilie Roger, Milad Pourbaghi-Masouleh, Laurent Lemaire, Clément Tétaud, Philippe Menei

**Affiliations:** a Département de Neurochirurgie, CHU, Angers, France;; b CRCINA, INSERM, Université de Nantes, Université d’Angers, Angers, France;; c MINT, INSERM 1066, CNRS 6021, Université d'Angers, UNIV Angers, Angers, France;; d Division of Drug Delivery and Tissue Engineering, School of Pharmacy, University of Nottingham, Nottingham, UK;; e PRISM-IRM, UNIV Angers, Angers, France

**Keywords:** Drug delivery, glioblastoma, lipid nanocapsules, sorafenib

## Abstract

Anticancer agents that target both tumor cells and angiogenesis are of potential interest for glioblastoma (GB) therapy. One such agent is sorafenib (SFN), a tyrosine kinase inhibitor. However, poor aqueous solubility and undesirable side effects limit its clinical application, including local treatment. We encapsulated SFN in lipid nanocapsules (LNCs) to overcome these drawbacks. LNCs are nanocarriers formulated according to a solvent-free process, using only components that have received regulatory approval. SFN-LNCs had a diameter of 54 ± 1 nm, high encapsulation efficiency (>90%), and a drug payload of 2.11 ± 0.03 mg/g of LNC dispersion. They inhibited *in vitro* angiogenesis and decreased human U87MG GB cell viability similarly to free SFN. *In vivo* studies showed that the intratumoral administration of SFN-LNCs or free SFN in nude mice bearing an orthotopic U87MG human GB xenograft decreased the proportion of proliferating cells in the tumor relative to control groups. SFN-LNCs were more effective than free SFN for inducing early tumor vascular normalization, characterized by increases in tumor blood flow and decreases in tumor vessel area. These results highlight the potential of LNCs as delivery systems for SFN. The vascular normalization induced by SFN-LNCs could be used to improve the efficacy of chemotherapy or radiotherapy for treating GB.

## Introduction

Glioblastoma (GB) is the most frequent, aggressive, and fatal type of brain tumor, with a mean 5-year survival rate of less than 5% (Stupp et al., 2005, 2009). Despite considerable scientific and technological progress, the treatment of GB remains a major challenge.

Signaling pathways initiated by activated receptor tyrosine kinases (RTKs), including those for epidermal growth factor (EGFR), platelet-derived growth factor (PDGFR), or vascular endothelial growth factor (VEGFR), play a key role in the growth, invasiveness, and angiogenesis of this tumor and are attractive therapeutic targets (Chen et al., 2016; Miller & Wen, 2016; Lin et al., 2017). Several drugs directed against RTK signaling pathways have been developed. Sorafenib (Nexavar™, SFN), approved for the treatment of advanced hepatocellular carcinoma, renal cell carcinoma, and thyroid cancer, is one such drug (Wilhelm et al., 2006). SFN is a multikinase inhibitor that acts on cell surface RTKs (e.g. VEGFR-2 and VEGRF-3, PDGFR-β, c-kit, and Flt-3) and downstream intracellular serine/threonine kinases (e.g. Raf-1, wild-type B-Raf, and mutant B-Raf). SFN has demonstrated anti-GB activity in both *in vitro* and *in vivo* models, inhibiting cell proliferation and angiogenesis (Siegelin et al., 2010; Yang et al., 2010; Carra et al., 2013). However, SFN treatment has been shown to be of very limited efficacy in patients with progressive or recurrent GB, either as a monotherapy or in combination with temozolomide or other targeted drugs, such as erlotinib (Reardon et al., 2011; Lee et al., 2012; Den et al., 2013; Peereboom et al., 2013; Zustovich et al., 2013; Hassler et al., 2014; Hottinger et al., 2014). This may be due, in part, to the route of SFN administration. The poor solubility of SFN strongly limits its application for local treatment and this drug is orally administered in the form of SFN tosylate tablets. This mode of administration may be effective for peripheral tumors, such as hepatocellular carcinoma, renal cell carcinoma, and thyroid cancer, but less so for brain tumors, due to the low efficiency of SFN passage across the blood-brain barrier (BBB) (Agarwal et al., 2011). High systemic doses of this drug are required to obtain effective brain-tumor concentrations, but the potential adverse events associated with the systemic administration of SFN, such as hand-foot skin reactions, rashes, upper and lower gastrointestinal distress, fatigue, and hypertension, rule out this mode of administration (Brose et al., 2014). Systems for delivering SFN for local or systemic treatment are, therefore, required, to improve the anti-GB activity of this drug. We recently showed that mesenchymal stromal cells (MSCs) have the potential to transport SFN to brain tumors following intranasal administration (Clavreul et al., 2017). However, the therapeutic effect was modest, probably due to the pro-tumorigenic properties of the MSCs themselves, which may counteract the action of the released SFN. The use of nanocarriers for the brain tumor delivery of SFN is another possibility. We studied lipid nanocapsules (LNCs) for SFN encapsulation because of the advantages they offer over other types of nanocarriers. These advantages include their production by a phase-inversion process using generally recognized as safe (GRAS) excipients, without the use of organic solvents, their high stability and drug loading capacity, and the possibility of scaling up their production easily (Huynh et al., 2009; Saliou et al., 2013). The direct intracranial delivery of drug-loaded LNCs by stereotaxic injection has yielded promising results in orthotopic GB models (Allard et al., 2008, 2009, 2010; Vinchon-Petit et al., 2010; Vanpouille-Box et al., 2011; Huynh et al., 2012; Balzeau et al., 2013; Danhier et al., 2015; Lollo et al., 2015; Cikankowitz et al., 2017; Séhédic et al., 2017). Furthermore, the LNC surface can be modified by incorporating specific molecules to improve the brain tumor targeting of these nanovectors (Béduneau et al., 2007; Laine et al., 2012; Balzeau et al., 2013; Hirsjärvi et al., 2014; Lollo et al., 2015; Séhédic et al., 2017).

We describe here the preparation of SFN-loaded LNCs (SFN-LNCs) and their characterization in terms of size, polydispersity index (PDI), surface charge, drug payload, *in vitro* drug release, and storage stability. Their toxicity against the U87MG GB cell line and effects on angiogenesis were evaluated *in vitro* and *in vivo*.

## Materials and methods

### Chemicals

SFN powder was purchased from LC Laboratories (Wobern, USA). Oil solubilizers and excipients were provided by Gattefosse S.A (Saint-Priest, France). Lipoid^®^ S75-3 (soybean lecithin with 70% phosphatidylcholine and 10% phosphatidylethanolamine) and Kolliphor^®^ HS15 (mixture of free polyethylene glycol 660 and polyethylene glycol 660 hydroxystearate) were gifts from Lipoid Gmbh (Ludwigshafen, Germany) and BASF (Ludwigshafen, Germany), respectively. NaCl was purchased from Prolabo VWR International (Fontenay-sous-Bois, France). Purified water was obtained with a MilliQ185 System (Millipore, Paris, France). Formic acid, acetic acid, acetonitrile, dimethylsulfoxide (DMSO), methanol, and HPLC-grade tetrahydrofurane (THF) were purchased from Sigma-Aldrich (Saint-Quentin Fallavier, France) and Carlo Erba reagents (Val-de-Reuil, France).

### Analytical methods

#### Analysis of SFN by high-performance liquid chromatography (HPLC-UV)

An HPLC-UV method was used to quantify SFN, as previously described (Clavreul et al., 2017). Briefly, HPLC was performed on a Waters modular system (600/717/996/2414) (Waters, Saint-Quentin-en-Yvelynes, France) with a SunFire^®^ C18 column (150 × 4.6 mm; 5 μm) at 37 °C. SFN was eluted with an isocratic mobile phase (acetonitrile/methanol/1% acetic acid, at a ratio of 35:38:27), at a flow rate of 1 mL/min, with monitoring at 266 nm. The chromatograms were recorded and integrated with Empower 3 software (Waters). The response was linear from 0.5 to 32 μg/mL.

#### Analysis of SFN by liquid chromatography tandem-mass spectrometry (LC-MS/MS)

A specific LC-MS/MS method was previously developed (Clavreul et al., 2017). Briefly, chromatography was performed on a Waters Alliance^®^ 2695 system with an Uptisphere^®^ 5 0DB (150 × 2.0 mm) column, at 25 °C, using an isocratic mixture of 0.1% formic acid in water/0.1% formic acid in acetonitrile: 20/80 (v/v) at a flow rate of 0.3 mL/min. Detection was performed using electrospray ionization in positive ion multiple reaction monitoring (MRM) mode, with the mass transition, m/z 465 → 270. Quantification was achieved with QuantLynx^®^ (Waters), by comparing the observed peak area ratios of SFN samples with a calibration curve obtained under the same experimental conditions. The calibration curve was linear in the concentration range of 50–1000 ng/mL.

### Solubility studies

A screening study was performed to determine the excipients likely to solubilize SFN. We added 10 mg SFN to 1 g excipient. Preparations were vortexed for 5 min, sonicated for 45 min, and centrifuged. Supernatants were extracted and analyzed using HPLC-UV.

### LNC formulation

SFN-LNCs were prepared according to the phase-inversion temperature method (Heurtault et al., 2002). SFN was first solubilized in Transcutol^®^ HP (0.7 g), by vortexing for 5 min. Labrafac^®^ WL1349 (0.4 g), Labrafil^®^ M1944CS (1 g) and Lipoïd^®^ S75-3 (150 mg) were then added. The compounds were heated on a hot plate at 80 °C, with shaking at 1200 rpm, until the Lipoïd^®^ S75-3 was completely dissolved. Once the mixture returned to room temperature, the other compounds of the formulation (i.e., water, NaCl, and Kolliphor^®^ HS15 (1.8 g, 0.1 g, and 1 g, respectively)) were introduced. Three cycles of progressive heating to 90 °C and cooling to 60 °C were then carried out, and, at 75 °C during the last cycle, an irreversible shock was induced by adding 5 mL of water at 0 °C. The LNC suspension was subjected to slow magnetic stirring for 5 min at room temperature. The formulation was filtered through a Minisart^®^ filter with 0.2 µm pores (Sartorius, Goettingen, Germany) and stored at 4 °C for further characterization. Blank LNCs (B-LNCs) were formulated in the same way but with the omission of SFN.

### LNC characterization

#### Particle size and zeta potential measurements

The size, PDI, and charge distribution of LNCs were measured using dynamic light scattering (DLS) on a Zetasizer^®^ Nano series DTS 1060 (Malvern Instruments S.A., Worcestershire, UK). The PDI was used to estimate the size distribution. A PDI value <0.2 indicates a unimodal size distribution. LNCs were diluted 1:60 (v/v) in deionized water, and three consecutive measurements were performed.

#### Encapsulation efficiency and drug loading

Drug loading was carried out using HPLC-UV, as described above. Three samples were prepared by dissolving 10 µL filtered SFN-LNCs in 990 µL 79/20 (v/v) methanol/THF and then filtering through a Millex-LG filter unit (0.2 µm pores). SFN loading was calculated and expressed in mg of SFN/g of LNC dispersion. Encapsulation efficiency (%) was determined by dividing the experimental drug loading by the theoretical drug loading.

#### Storage stability studies

The stability of SFN-LNC formulations (*n* = 4) was evaluated after storage at 4 °C for 4 months. The particle size, PDI, zeta potential, and drug payload of the samples were determined after filtration through a Minisart^®^ filter with 0.2 µm pores (Sartorius), as described above.

#### Evaluation of drug-release profile

SFN-LNCs (*n* = 4) were diluted 1:170 (v/v) in DPBS (Ozyme, St Quentin-en-Yvelines, France) and incubated at 37 °C with shaking. A 1 mL release medium sample was withdrawn at intervals of 0.5, 1, 2, 3, 8, 24, 48, and 120 h and replaced with 1 mL DPBS. Samples were filtered through a Minisart^®^ filter with 0.2 µm pores (Sartorius), to remove free precipitated SFN. The cumulative release of SFN from LNCs was determined indirectly, by evaluating SFN loading by the LC-MS/MS method.

### In vitro studies

#### Cell culture

The human GB cell line U87MG was obtained from the ATCC (LGC Promochem, Molsheim, France) and expanded in DMEM-high glucose medium (DMEM-HG, Ozyme) containing 10% FBS (Fisher Scientific, Illkirch, France) and 1% antibiotics (Sigma-Aldrich). Human umbilical vein endothelial cells (HUVECs) were purchased from Lonza (Verviers, Belgium). Cells were cultured, according to the supplier’s instructions, in endothelial cell growth medium-2 (EGM-2), corresponding to endothelial basal medium-2 (EBM-2) containing the supplements and growth factors of the EGM-2 SingleQuot™ kit (Lonza). U87MG cells and HUVECs were maintained under an atmosphere containing 5% CO_2_ (37 °C), in a humidified incubator, until they reached 80% confluence.

#### U87MG viability assay

U87MG (5 × 10^3^ cells/cm^2^) were plated in 96-well plates. After 48 h, the culture medium was removed, and the cells were treated with SFN and SFN-LNCs, at concentrations of 0.001–100 µM. B-LNCs were also tested with the same excipient concentration. After 96 h, the medium was removed and the plates were stored at −80 °C until the assays were carried out. Cell survival was estimated with the CyQUANT^®^ cell proliferation assay kit, according to the manufacturer’s instructions (Fisher Scientific).

#### Endothelial cell tube-formation assay

HUVECs (5 × 10^4^ cells/cm^2^) were incubated in Corning^®^ Matrigel^®^ basement membrane matrix-coated 96-well plates (VWR International, Fontenay-sous-Bois, France) for 16 h in EGM-2, with or without SFN, B-LNCs, or SFN-LNCs. The endothelial tubes formed were photographed and quantified. The degree of tube formation was assessed by manual counting of the number of tube-like structures in four wells for each set of conditions.

### *In vivo* studies

A scheme for the protocol used in this study is presented in Figure S1.

**Figure 1.**
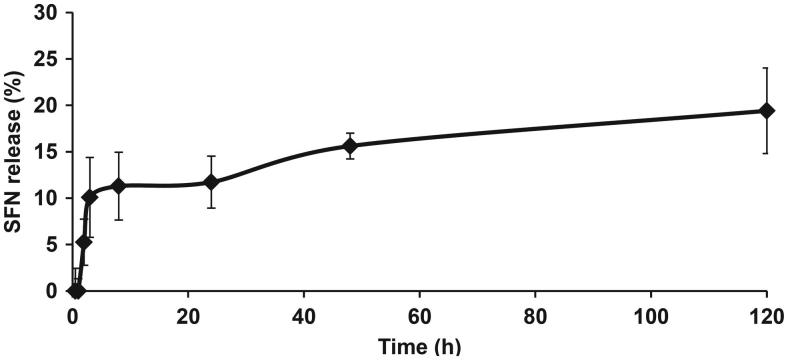
SFN release profile from LNCs in DPBS (*n* = 4).

#### Intratumor administration of SFN-LNCs in the U87MG GB model

Female Swiss nude mice (8–10-weeks old) were obtained from Charles River Laboratories (L’Arbresle, France). The protocol was approved by the Committee for the Ethics of Animal Experiments of the ‘Pays de la Loire’ (Permit no. 01785.01). Animals were anesthetized by an intraperitoneal injection of xylazine (13 mg/kg body weight) and ketamine (100 mg/kg body weight) and positioned in a Kopf stereotaxic instrument. On day 0, U87MG cells (5 × 10^4^) in 5 μL HBSS containing Ca^2+^ and Mg^2+^ were injected into the right striatum of the mice [coordinates: 2.1 mm lateral to the bregma and 0.5 mm anterior and 3 mm interior to the outer border of the cranium]. On day 9, the mice were assigned to four groups and received an injection, by convection-enhanced delivery (CED) (5 µL; 0.5 µL/min) at the same coordinates of (a) HBSS with Ca^2+^ and Mg^2+^ (*n* = 5); (b) B-LNCs (*n* = 5); (c) SFN-LNCs (*n* = 7, 3.5 µg); or (d) SFN (*n* = 5, 3.5 µg). Tumor volume and perfusion were measured on day 13. Mice were killed on day 16, and the number of proliferating Ki67^+^ cells and the area of CD31^+^ vessels in the U87MG tumor was analyzed. Brains were snap-frozen in liquid nitrogen-cooled isopentane and stored at −80 °C. Coronal sections of the brain were cut at 10 µm intervals and collected on silane-treated slides. For histological analysis, ethanol (95%)/acetic acid (5%)-fixed brain sections were stained with Mayer’s hematoxylin solution and permanently mounted.

#### *In vivo* measurement of brain tumor volume and perfusion

MRI was performed with a 7T scanner (Biospec 70/20 Avance III, Bruker, Wissembourg, France), equipped with a BGA12S gradient system (675 mT/m), under isoflurane anesthesia (1.5–0.5%, O_2_: 0.5 L/min). Body temperature was maintained at 36.5–37.5 °C with a feedback-regulated heating pad during the entire imaging protocol. Tumor volume was assessed over time with a 1H cryoprobe and rapid acquisition with a relaxation enhancement (RARE) sequence [TR = 3200 ms; effective echo time (TEeff) = 21.3 ms; acceleration factor = 4; FOV = 2 × 2 cm; matrix 256 × 256; 11 contiguous slices of 0.5 mm, Nex = 1]. Volumes were calculated from manually drawn regions of interest (ROI). Tumor perfusion was assessed by segmented fast imaging with a steady-state precession arterial spin labeling sequence (FISP-ASL), as previously described (Lemaire et al., 2016). Homogeneous radiofrequency excitation was achieved using a proton volume resonator (diameter 87 mm, homogeneous length 80 mm) and signal reception was performed with an actively decoupled phased-array surface coil (4 channels). Blood flow was measured from two T1 maps acquired once with slice-selective inversion and once with global inversion (Kober et al., 2004). A series of 40 gradient echoes were acquired after the inversion pulse to acquire T1 maps (flip angle = 8°, echo time = 1.8 ms, field of view = 18 × 18 mm, matrix size = 128 × 128, excitation hermite pulse duration = 800 μs, inversion hyperbolic secant pulse duration = 15 ms, imaging slice thickness = 1.5 mm, labeling slice thickness = 3.9 mm, with the first echo started 20 ms after the inversion pulse, and a 60 ms interval among echoes). Thirty-two segments were used to fill *k*-space. A repetition delay of 13 s was introduced after the acquisition of a set of gradient echoes to allow for full relaxation between two inversion pulses. The total measurement time was approximately 14 min. ROIs were manually outlined in the tumor core, the surrounding tissue, and out to the contralateral side of the brain for comparison. Blood flow in these ROIs was calculated using ParaVision 5.1 software (Bruker).

#### Immunofluorescence

For CD31 and Ki67 expression analyses, brain cryosections were allowed to dry in air, rehydrated in DPBS, and fixed by incubation for 10 min in 4% PFA, pH 7.4, at 4 °C. Nonspecific binding was blocked by incubating the sections in 4% BSA and 10% normal goat serum in DPBS. The sections were incubated overnight, at 4 °C, with isotype controls and primary antibodies against endothelial cells (mouse CD31, BD Biosciences, Le Pont de Claix, France) and proliferative cells (Ki67, Agilent Technologies, Les Ulis, France). The primary antibodies were detected with biotinylated secondary antibodies, and the signal was amplified with Alexa Fluor 488 streptavidin (Interchim, Montluçon, France). Nuclei were counterstained with DAPI (Sigma-Aldrich). Cryosections of four mice from each group described above (a, b, c, and d) were analyzed under an Axioscope^®^ 2 fluorescence microscope. CD31^+^ and Ki67^+^ cells were counted with the MetaView computerized image-analysis system in six brain cryosections per mouse, corresponding to three areas of the tumor approximately 400 µm apart (Figure S1). Five fields per cryosection, at ×200 magnification, were randomly selected for each tumor.

### Statistics

Results are expressed as the mean ± SEM (standard error of the mean). The Mann–Whitney *U*-test and one-way ANOVA, followed by Dunnett’s post hoc test for multiple comparisons were used for statistical analyses. Differences were considered to be significant if the *p*-value was <.05.

## Results

### Production of SFN-LNCs

#### Studies of SFN solubility

SFN solubility in six different excipients (five oils and one co-surfactant), potentially useful for LNC formulation, was analyzed at 1% (w/w). SFN was soluble only in Transcutol^®^ HP, at a maximum rate of 120 mg/g (Table 1).

**Table 1.** Solubility of SFN in various excipients at 1% (w/w).

Excipients	Function	Solubility
Transcutol^®^ HP	Co-surfactant	S
Labrafil^®^ M1944CS	Oil	NS
Peceol™	Oil	NS
Labrafac^®^ WL1349	Oil	NS
Captex^®^ 8000	Oil	NS
Oleic acid ≥99%	Oil	NS

S: Soluble; NS: not soluble.

#### Formulation of SFN-LNCs

Based on the solubility of SFN in Transcutol^®^ HP and the results of Roger et al. (2011), a mixture of Labrafac^®^ WL1349, Labrafil^®^ M1944CS, and Lipoïd^®^ S75-3 was used to formulate LNCs. B-LNCs had a mean diameter of 49 ± 1 nm and narrow size distribution (PDI = 0.11) (Table 2).

**Table 2.** Characterization of B-LNCs (*n* = 6) and SFN-LNCs (*n* = 17).

Sample	Size (nm)	PDI	Zeta potential (mV)	Drug payload (mg/g)	Encapsulation efficiency (%)
B-LNCs	49 ± 1	0.11 ± 0.01	−7.9 ± 0.4	–	–
SFN-LNCs	54 ± 1	0.15 ± 0.01	−7.8 ± 0.6	2.11 ± 0.03	105 ± 1

We assessed the physicochemical characteristics of SFN-LNCs (filtration ability, peak number and PDI) as a function of the amount of SFN (15, 20, 30, 50, and 84 mg) dissolved in Transcutol^®^ HP (0.7 g). The formulations became increasingly difficult to filter as the quantity of SFN increased, and particle size became more heterogeneous and the range of particle sizes broader (Table S1). We set the amount of SFN used in the formulation to 20 mg on the basis of these observations. The resulting SFN-LNCs had physicochemical properties similar to those of B-LNCs and a drug payload of 2.11 mg/g of LNC dispersion with an encapsulation efficiency >90% (Table 2).

#### In vitro release profile

We then assessed the percentage of SFN released from SFN-LNCs into DPBS buffer over time (Figure 1). We observed a burst of SFN release within the first 8 h, the dose reaching ∼11% of the total SFN. Subsequently, SFN was released in a gradual and sustained manner: ∼20% of the initial dose within 120 h.

#### Storage stability

SFN-LNCs were physically stable at 2–8 °C for at least four months (Table S2). We observed no meaningful change in mean LNC size, pH, or zeta potential. In addition, there was no variation in drug payload after four months.

### In vitro and in vivo effects of SFN-LNCs

#### Effect of SFN-LNCs on U87MG tumor cells and endothelial tube formation

**Figure 2:**
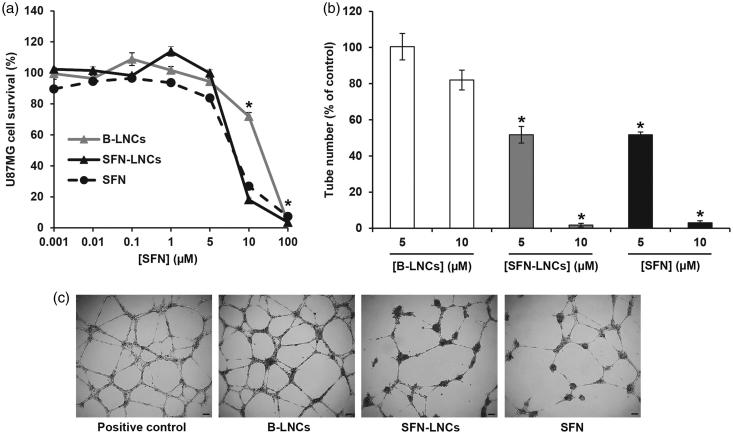
Effect of SFN-LNCs on U87MG tumor cell viability and the ability of HUVECs to form tube-like structures. (a) U87MG cell viability following exposure to various concentrations of B-LNCs, SFN-LNCs, or SFN (0.001–100 μM). Data are expressed as the means ± SEM (*n* = 4). The results obtained for U87MG cells grown in culture medium alone were considered to correspond to 100% survival (*p < .05 for B-LNCs, SFN-LNCs, or SFN vs. culture medium alone, Mann-Whitney *U*-test). (b and c) Formation of tube-like structures by HUVEC following treatment with B-LNCs, SFN-LNCs, or SFN (5 or 10 μM). (b) Angiogenesis was quantified by manually counting the number of tube-like structures. Results are presented as means ± SEM. The number of tube-like structures obtained in EGM-2 alone was considered to correspond to 100% (*p < .05 for SFN-LNCs or SFN vs. EGM-2, Mann–Whitney *U*-test). (c) Representative phase-contrast micrographs of HUVEC tube formation on Matrigel 16 h after treatment with 5 µM B-LNCs, SFN-LNCs, or SFN. The positive control corresponded to EGM-2 medium alone (scale bar = 100 µm).

Neither free SFN nor SFN-LNCs altered the growth of U87MG cells at concentrations below 5 µM (Figure 2(a)). U87MG cell survival decreased at concentrations above 5 µM, corresponding to an IC_50_ of 7.39 ± 016 µM for free SFN and 7.56 ± 0.07 µM for SFN-LNCs. The viability of U87MG was unaltered by formulation in B-LNCs, except at the highest concentration tested. We evaluated the antiangiogenic properties of SFN-LNCs in a HUVEC tube-formation assay (Figure 2(b,c)). Treatment with B-LNCs did not affect EGM-2-induced tube formation, whereas incubation with 5 or 10 µM SFN-LNCs or SFN markedly decreased tube formation, in a dose-dependent manner (Figure 2(b,c)).

#### Evaluation of the intratumoral administration of SFN-LNCs in the orthotopic U87MG GB model

We assessed the effect of intratumoral CED infusion of SFN-LNCs on U87MG growth and angiogenesis, as described in Figure S1.

**Figure 3.**
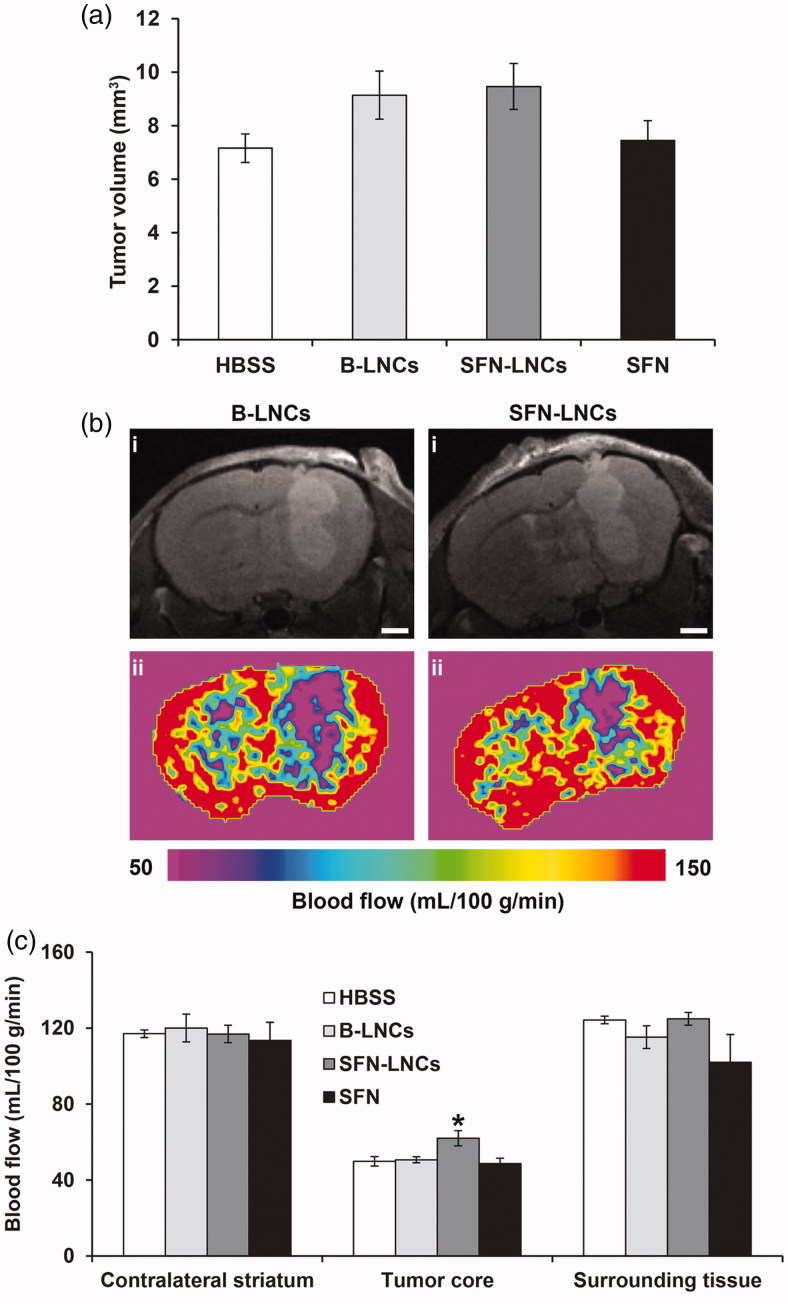
Effect of SFN-LNCs on tumor volume and perfusion in U87MG-bearing mice 4 days after treatment (D13). (a) Tumor volume distribution in each group, calculated from MRI images. (b) Perfusion MRI images of B-LNC- and SFN-LNC-treated U87MG glioma-bearing mice (scale bar = 1 mm). T2-weighted morphological images are shown in the top panels (i) and perfusion maps in the bottom panels (ii). (c) Graph showing blood-flow values in the tumor core, the surrounding tissue, and the contralateral striatum. Blood flow (mL/100 g/min) was measured by the ASL perfusion MRI method (*p <.05 for SFN-LNCs vs. HBSS, one-way ANOVA followed by Dunnett’s post hoc test for multiple comparisons).

***Tumor volume alterations.*** There was no significant difference in tumor volume between control vehicle-treated groups (HBSS and B-LNCs) and SFN-LNC- or SFN-treated groups 4 days after treatment (D13) (HBSS mean = 7.2 ± 0.6 mm^3^, B-LNC mean = 9.1 ± 0.9 mm^3^, SFN-LNC mean = 9.5 ± 0.9 mm^3^, SFN mean = 7.4 ± 0.7 mm^3^) (Figure 3(a)). Control vehicle-, SFN-LNC-, and SFN-treated-tumors had similar growth rates between MRI (D13) and histological and immunofluorescence analyses (D16) (data not shown).

***Tumor perfusion analyses.*** We determined tumor perfusion from ASL-MRI perfusion maps, to assess the tumor microvasculature. In all animals, tumor blood flow (TBF) was weaker than that in the surrounding tissue or contralateral brain tissue (Figure 3(b,c)). Blood flow in the tumor core of mice treated with SFN-LNCs was slightly stronger than that in the tumor core in control vehicle-treated mice and SFN-treated mice (Figure 3(b,c)) (HBSS mean = 50 ± 3 mL/100 g/min, B-LNC mean = 51 ± 2 mL/100 g/min, SFN-LNC mean = 62 ± 4 mL/100 g/min, SFN mean = 49 ± 3 mL/100 g/min, *p* < .05: SFN-LNC vs. HBSS).

**Figure 4.**
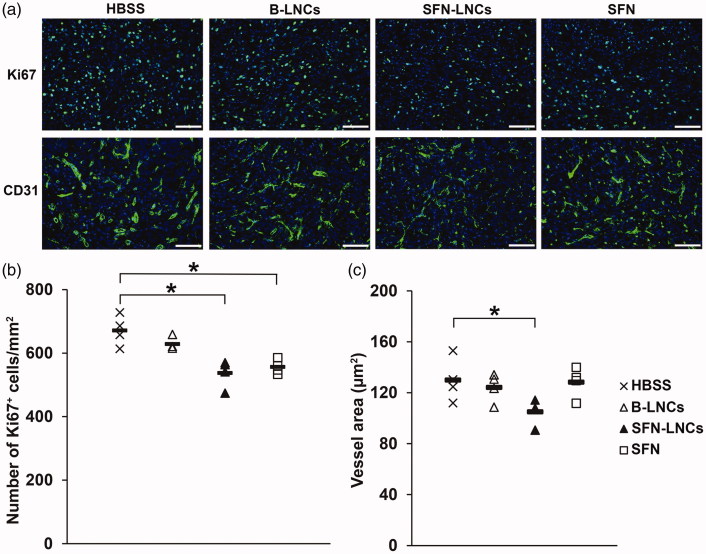
Effect of SFN-LNCs on Ki67^+^ cell number and CD31^+^ vessel area. (a) Immunofluorescence staining for Ki67 and CD31 in the tumor on day 16 in each group of animals (scale bar = 100 μm). (b and c) Quantitative results for Ki67 and CD31 immunofluorescence. Results are expressed as the mean number of Ki67^+^ cells per mm^2^ ± SEM (b) or CD31^+^ vessel area ± SEM (c) (*p < .05 vs. HBSS, one-way ANOVA followed by Dunnett’s post hoc test for multiple comparisons).

***Analyses of the immunofluorescence of intratumoral Ki67^+^ cells and CD31^+^ vessels*.** SFN-LNC or SFN treatment decreased the number of intratumoral Ki67^+^ cells in U87MG-bearing mice relative to control vehicle-treated mice 7 days after administration (Figure 4(a,b)) (HBSS mean = 671 ± 24 Ki67^+^ cells/mm^2^, B-LNC mean = 629 ± 10 Ki67^+^ cells/mm^2^, SFN-LNC mean = 537 ± 22 Ki67^+^ cells/mm^2^, SFN mean = 556 ± 11 Ki67^+^ cells/mm^2^, *p* < .05: SFN-LNC or SFN vs. HBSS). SFN-LNC treatment resulted in a smaller tumor vessel area than HBSS, B-LNC, or free SFN treatment (Figure 4(a,c)) (HBSS mean = 130 ± 9 µm^2^, B-LNC mean = 124 ± 6 µm^2^, SFN-LNC mean = 105 ± 5 µm^2^, SFN mean = 128 ± 6 µm^2^, *p* < .05: SFN-LNC vs. HBSS). Control vehicle-, SFN-LNC-, and SFN-treated animals had similar numbers of tumor vessels (data not shown).

## Discussion

SFN has been shown to be a potential targeted molecular therapeutic agent for various solid tumors, including GB. It targets RTKs and the RAF/MEK/ERK pathway, thereby acting as a combined antiangiogenic and antitumorigenic drug. However, poor solubility in water and undesirable side effects limit the clinical applications of SFN, even for local treatment. New strategies are required to overcome these drawbacks. Nanoformulations of SFN are of potential interest, as they combine the intrinsic toxicity of the drug with a nanodelivery approach. Many studies have assessed the use of SFN encapsulated or entrapped in various nanocarriers, including polymeric carriers (Zhang et al., 2013; Craparo et al., 2014; Gao et al., 2015; Li et al., 2015; Liu et al., 2015, 2016; Lin et al., 2016; Yang et al., 2016), lipid-based carriers (Zhang et al., 2014; Bondì et al., 2015; Grillone et al., 2015; Liu et al., 2015; Xiao et al., 2016; Yang et al., 2016; Mo et al., 2018; Benizri et al., 2018), and polymer-lipid hybrid nanoparticles (Zhang et al., 2016, 2017). These nanocarriers have given promising results in the liver and gastric cancer models. However, most of these formulations have major drawbacks, such as the presence of organic solvents or toxic compounds, preventing their clinical use. Here, we encapsulated SFN in solvent-free LNCs, which were produced exclusively from GMO-free and GRAS excipients (Hureaux et al., 2009, 2010; Le Roux et al., 2017).

We determined the solubility of SFN in various LNC formulation constituents. Only Transcutol^®^ HP was able to dissolve SFN. As this compound is a co-surfactant, Labrafac^®^ WL1349 and Labrafil^®^ M1944CS were added to constitute the oily phase of the formulation. SFN-LNCs have a PDI <0.2, indicating monodispersity, and a mean particle size of 54 ± 1 nm. The surface charge on SFN-LNCs was negative, favoring the dispersion of LNCs rather than their aggregation. The encapsulation efficiency of SFN-LNCs was >90%, indicating that the SFN was almost completely entrapped within the LNCs. SFN-LNCs were stable for over 4 months at 4 °C, with no change in characteristics. After 5 days in DPBS buffer, approximately 20% of the SFN was released. This slow-release profile suggests that most of the SFN remained associated with the LNCs, which may be beneficial, as it would allow sufficient time for cells to capture the loaded LNCs. High encapsulation efficiency and slow drug release have also been reported for liposomal formulations of SFN (Liu et al., 2015; Mo et al., 2018).

SFN-LNCs had cytostatic effects against U87MG tumor cells and endothelial cells similar to those of free SFN, suggesting that SFN activity is conserved during LNC encapsulation. We evaluated the *in vivo* effect of SFN-LNCs in nude mice bearing an orthotopic human U87MG GB xenograft, after their intratumoral administration by CED. CED has the advantage of bypassing the BBB and results in a greater volume of distribution than a bolus injection (Ndesendo, 2015). The combination of LNCs with the CED technique has yielded promising results in orthotopic GB models for the delivery of therapeutic agents, such as lipophilic complexes of rhenium-188 (^188^Re-SSS) (Allard et al., 2008; Vanpouille-Box et al., 2011; Cikankowitz et al., 2017; Séhédic et al., 2017), ferrociphenol (Allard et al., 2009, 2010; Huynh et al., 2012), paclitaxel (Vinchon-Petit et al., 2010; Balzeau et al., 2013; Lollo et al., 2015), and anti-EGFR and anti-Galectin-1 siRNAs (Danhier et al., 2015). We were, particularly, interested in detecting early vascular changes in response to SFN-LNCs, as endothelial cells are highly sensitive to SFN. We, thus, performed ASL-MRI, a noninvasive and quantitative technique that measures perfusion by magnetically labeling water as a freely diffusible endogenous tracer (Silva et al., 2000), on large established U87MG tumors (approximately 8 mm^3^). This technique has already been used in other studies, to evaluate early antiangiogenic treatment responses for brain tumors in humans (Fellah et al., 2011; Andre et al., 2015) and rodents (Rajendran et al., 2014; Towner et al., 2015; Yun et al., 2016; Ziegler et al., 2017). Blood flow in the tumor core was weaker than in normal brain tissue, consistent with the results of Sun et al. (2004). Such an impairment of blood flow is frequently described in GB, due to a dilated, tortuous, disorganized, and leaky vasculature (Boucher et al., 1996; Jain, 2001; Chauhan et al., 2011). Four days after treatment, perfusion in the tumor core of animals treated with SFN-LNCs was greater than in control animals and animals treated with SFN alone. The SFN-LNC-treated group had a TBF value of 62 ± 4 mL/min/100 g, whereas the HBSS control group had a TBF value of 50 ± 3 mL/min/100 g. This increase in blood flow was localized exclusively to the tumor core. Blood flow was not altered in the surrounding tissue or in the contralateral striatum. TBF is known to be dependent on tumor size, regardless of the type of solid tumor (Kallinowski et al., 1989; Hwang et al., 2015; Lemaire et al., 2016). Lemaire et al. (2016) observed a decrease in TBF of about 3 mL/min/100 g for every 1 mm^3^ increase in tumor volume. Mean tumor size was 9.5 ± 0.9 mm^3^ and 7.2 ± 0.5 mm^3^ for the LNC-SFN-treated and HBSS control groups, respectively. Thus, the increase in TBF observed in the LNC-SFN-treated group was not due to a decrease in tumor volume. In addition to the increase in perfusion with SFN-LNC treatment, we observed a decrease in tumor vessel area. These data suggest that the SFN-LNCs may have normalized abnormal vessel structures, potentially leading to an increase in perfusion. Vessel normalization following treatment with SFN or other RTK inhibitors has already been described for various cancers (Wilhelm et al., 2006; Sorensen et al., 2012; Batchelor et al., 2007, 2013; Navis et al., 2013). We observed no such vascular changes following the treatment of U87MG-bearing mice with free SFN, highlighting the potential of SFN-LNCs. This may be due to the capacity of LNCs to improve SFN retention within the tumor. The prolonged retention of LNCs in brain tumors following intratumoral CED administration was previously reported in a study of LNCs loaded with ^188^Re-SSS (LNC^188^Re-SSS) (Vanpouille-Box et al., 2011; Cikankowitz et al., 2017). Most (70%) of the ^188^Re-SSS activity was present in the tumor region 24 h after LNC ^188^Re-SSS injection, whereas free ^188^Re-perrhenate was rapidly eliminated in the urine and feces. In addition to inducing early vascular changes, SFN-LNC treatment reduced the number of proliferative Ki67^+^ cells in the tumor. We also observed this effect after treatment with free SFN, probably due to the faster uptake of free SFN by tumor cells than by endothelial cells. Despite the increase in tumor perfusion and the decreases in tumor vessel area and the number of Ki67^+^ cells achieved with SFN-LNCs, these effects were not sufficient to modify the growth rate of U87MG tumors. It should be noted that we treated large established U87MG tumors, in this study, to allow perfusion analyses, which require a tumor thickness of at least 1.5 mm. This condition may not be compatible with the achievement of a therapeutic effect, given the aggressiveness of U87MG tumors (median survival of approximately 24 days following the intrastriatal administration of 5 × 10^4^ U87MG cells). Furthermore, the dose of SFN-LNCs injected may not be sufficient to modify U87MG tumor growth. Siegelin et al. (2010) observed that the daily treatment of U87MG-bearing mice with SFN (100 mg/kg) administered by intraperitoneal injection resulted in an inhibition of tumor-cell proliferation and lower levels of angiogenesis, resulting in prolonged survival in mice. We injected only one dose of SFN-LNCs (3.5 µg/mouse), a lower dose than that used in the study by Siegelin et al., corresponding to about 2 mg/mouse/day. A repeated-injection regimen is probably required for SFN-LNCs, to have an effect on U87MG tumor growth. However, CED is an invasive method, making repeated treatment difficult. In a clinical context, the systemic administration of SFN-LNCs would be simpler, less costly, and more compatible with long-term treatment. The intravenous route is the route most frequently used for LNC administration (Huynh et al., 2011; Hirsjärvi et al., 2014; Lainé et al., 2014). Owing to the nonspecific uptake of LNCs by the mononuclear phagocyte system and the presence of the BBB, obtaining an effective response to treatment following the systemic delivery of drug-loaded LNCs remains challenging (Huynh et al., 2009; Roger et al., 2011; Karim et al., 2016). Strategies for improving vascular function in GB have been reported to provide a window of opportunity for enhancing the efficacy of chemotherapy or radiotherapy (Jain, 2005). For example, the delivery of temozolomide in a preclinical intracerebral model of glioma increased after treatment with the angiogenesis inhibitor SU5416, which restored the capillary architecture (Ma et al., 2003). Wang et al. (2015) showed that GB-bearing mice treated with irinotecan in combination with bevacizumab, an antibody against VEGF, survived longer than those treated with irinotecan alone. Thus, SFN-LNCs could be used to improve the efficacy of other chemotherapy treatments.

## Conclusion

We demonstrate here the successful encapsulation of SFN in LNCs by a phase-inversion process, without the use of organic solvents. SFN-LNCs displayed cytotoxic activity against human U87MG GB cells and endothelial cells *in vitro*, similar to that observed with free SFN. The intratumoral CED administration of SFN-LNCs to U87MG-bearing mice decreased the proportion of proliferating cells and induced an early increase in tumor blood flow, associated with a decrease in tumor vessel area consistent with the induction of a vascular normalization process. The induction of this process by SFN-LNCs suggests that these nanocarriers could potentially be used to enhance the efficacy of chemotherapy or radiotherapy for treating GB. Further studies are required to evaluate and optimize dosing and schedule for effective SFN-LNC combinations with other drugs or radiotherapy.

## Supplementary Material

Table_S2.doc

Table_S1.doc

Figure S1
