# Supplementary material for: Development and characterization of sorafenib-loaded lipid nanocapsules for the treatment of glioblastoma
Source: Drug Deliv. 2018 Oct 19;25(1):1756–65. doi: 10.1080/10717544.2018.1507061 (PMC6225440; doi:10.1080/10717544.2018.1507061)
Supplement: Table_S2.doc [file IDRD_A_1507061_SM1007.doc]

**Table S2:** Stability data of SFN-LNCs stored at 2-8°C (size, polydispersity, zeta potential, and drug payload) (n = 4)

| **Time**  **(day)** | **Size**  **(nm)** | **PDI** | **Zeta potential (mV)** | **Drug payload (mg/g)** |
| --- | --- | --- | --- | --- |
| 0 | 54 ± 2 | 0.14 ± 0.01 | -7.4 ± 0.6 | 2.09 ± 0.03 |
| 15 | 53 ± 2 | 0.14 ± 0.01 | -9.7 ± 1.0 | 2.24 ± 0.04 |
| 30 | 54 ± 2 | 0.13 ± 0.01 | -9.0 ± 1.5 | 2.19 ± 0.01 |
| 60 | 55 ± 6 | 0.13 ± 0.01 | -6.9 ± 0.6 | 2.24 ± 0.02 |
| 120 | 51 ± 2 | 0.13 ± 0.01 | -9.7 ± 1.7 | 2.22 ± 0.06 |
