# Supplementary material for: Development and characterization of sorafenib-loaded lipid nanocapsules for the treatment of glioblastoma
Source: Drug Deliv. 2018 Oct 19;25(1):1756–65. doi: 10.1080/10717544.2018.1507061 (PMC6225440; doi:10.1080/10717544.2018.1507061)
Supplement: Table_S1.doc [file IDRD_A_1507061_SM1006.doc]

**Table S1:** Optimization of SFN formulation

| **SFN quantity (mg)** | **Filtration** | **Peak number** | **Size** | **PDI** |
| --- | --- | --- | --- | --- |
| 15 | Good | 1 | 44 | 0.07 |
| 20 | Good | 1 | 52 | 0.14 |
| 30 | Difficult | 2 | / | / |
| 50 | Difficult | 2 | / | / |
| 84 | Difficult | 1 | 99 | 0.23 |
