# Supplementary figures and images for: Development and characterization of sorafenib-loaded lipid nanocapsules for the treatment of glioblastoma
Source: Drug Deliv. 2018 Oct 19;25(1):1756–65. doi: 10.1080/10717544.2018.1507061 (PMC6225440; doi:10.1080/10717544.2018.1507061)

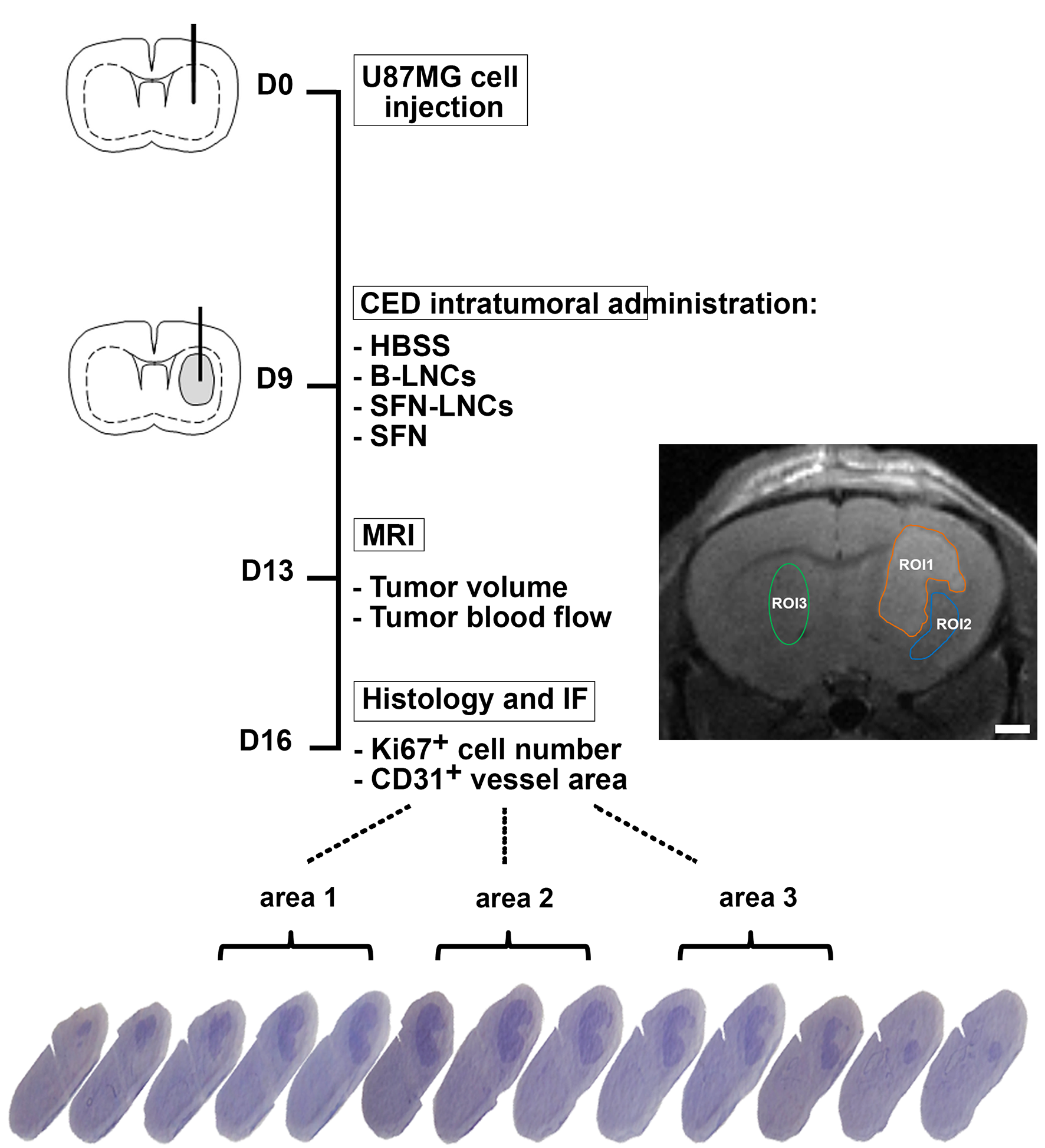

Supplement: Figure S1 [file IDRD_A_1507061_SM1005.tif]
